# Negotiating Intersecting Precarities: COVID-19, Pandemic Preparedness and Response in Africa

**DOI:** 10.1080/01459740.2021.2015591

**Published:** 2022-01-07

**Authors:** Hayley MacGregor, Melissa Leach, Grace Akello, Lawrence Sao Babawo, Moses Baluku, Alice Desclaux, Catherine Grant, Foday Kamara, Fred Martineau, Esther Yei Mokuwa, Melissa Parker, Paul Richards, Kelley Sams, Khoudia Sow, Annie Wilkinson

**Affiliations:** a Institute of Development Studies, University of Sussex, Brighton UK; b Department of Mental Health, Gulu University, Gulu, Uganda; c Njala University, Bo, Sierra Leone; d Institut de Recherche pour le Développement (IRD), Montpellier France; e Department of Global Health and Development, London School of Hygiene and Tropical Medicine, UK; f Infectious Diseases Department, CRCF, Dakar, Senegal

**Keywords:** Preparedness, COVID-19, Africa, epidemic, precarity, response

## Abstract

This article shares findings on COVID-19 in Africa across 2020 to examine concepts and practices of epidemic preparedness and response. Amidst uncertainties about the trajectory of COVID-19, the stages of emergency response emerge in practice as interconnected. We illustrate how complex dynamics manifest as diverse actors interpret and modify approaches according to contexts and experiences. We suggest that the concept of “intersecting precarities” best captures the temporalities at stake; that these precarities include the effects of epidemic control measures; and that people do not just accept but actively negotiate these intersections as they seek to sustain their lives and livelihoods.

The COVID-19 pandemic continues to have marked effects on health systems, economies, and livelihoods across the globe. Such impacts have been particularly acute in contexts that were already facing societal challenges such as austerity and deepening inequalities, a situation that has been characterized as one of “intersecting crises” (Dowler 2020). The focus of this article is the experience of COVID-19 in sub-Saharan Africa across 2020, especially for people living in two rural districts in Sierra Leone and Uganda, where preemptive government measures to contain viral spread compounded preexisting uncertainties in health, livelihoods, and citizen–state relations. Under such circumstances, COVID-19 has intensified precarity, both as a state of chronic insecurity and a political process of exclusion that disproportionately erodes conditions of life for those at the margins (Butler 2004, 2010). We propose the notion of “intersecting precarities” to encapsulate this reality of COVID-19 manifesting amidst already precarious lives and a paucity of state welfare, prompting people to take individual and collective action to negotiate the effects of public health restrictions. COVID-related measures, enforced within an acute emergency framework oriented toward averting a health crisis, were instituted in contexts already beset by chronic circumstances of uncertainty and “slow emergencies” (Anderson et al 2019).

In this context, the tensions that arise between different priorities, temporal framings, forms of agency, and relations of authority are of particular concern. Furthermore, we contend that the unfolding of the pandemic experience in sub-Saharan Africa challenges prevailing understandings of pandemic preparedness as a set of technical strategies oriented toward strengthening formal responses to anticipated future outbreaks. Rather, conditions of intersecting precarities point to the limitations of technical understandings of preparedness and the need to incorporate attention to political economy and the fragility of systems and lives, compounded by historical and ongoing processes of structural violence. The structural underpinnings of preparedness also emerge as critical to shaping the possibilities for response, collapsing a clear distinction between the two.

Recent years have seen increased attention to epidemic and pandemic preparedness in global health fora, with international agencies such as the World Health Organization (WHO) elaborating a largely technical, crisis-oriented approach focusing on surveillance systems, scenario planning, priority pathogen roadmaps and Joint Evaluation Exercises (JEEs) for readiness assessments. In 2016 the establishment of the WHO Health Emergencies Programme resulted in further elaboration of a phased epidemic emergency cycle, conceptualized as progressive temporal stages – prevention, preparedness, early warning, response, recovery.

There has been considerable social science attention to the emergence of preparedness as a distinctive paradigm to approach infectious disease risks (Caduff 2015; Lakoff 2008, 2017). This is associated with the rise of a global security framing of health threats, and an “anticipatory imagination” and alertness to predict outbreaks, instantiated through specific practices (Lakoff 2017). The structures, meanings, practices, and power relations that are mobilized have been conceptualized as “preparedness assemblages” (eg. Lakoff 2017; Samimian-Darash 2009) after Ong and Collier (2005). Preparedness has also been analyzed as a social process, the strength of the assemblages dependent on the material and relational infrastructures that can be harnessed and maintained (Lee et al. 2020). Critical analyses of past preparedness and response efforts argue that standard models privilege the technical and legal over social and ethical concerns (Garoon and Duggan 2008); underplay the significance of diverse forms of uncertainty (Leach et al. 2021); focus on emergency events in isolation from vital longer-term health system capacities (Farmer 2014); and can over-prioritize global systems, misdirecting attention and resources from the more pressing priorities of people who are vulnerable to outbreaks (Lachenal 2014; Nguyen 2014). The latter observation is prescient, pointing to a need for ethnographic attention to people’s own experiences of and responses to outbreaks in countries labeled as so-called “hotspots” for disease emergence, and thus the focus of preparedness efforts. Such accounts proliferated for Ebola in West Africa, suggesting *inter alia* instances where collective responses in villages drawing on local knowledge, social relations, trusted public authorities, and cultural logics contributed to outbreak containment (Abramowitz 2017; Parker et al. 2019; Richards 2016). Ethnographic attention may challenge formal preparedness assemblages and their narrow assumptions about data for containment (eg. Erikson 2018), “hotspots” and transmission patterns (eg. Brown and Kelly 2014).

Building on these insights, our team began research in January 2019 on contemporary meanings and practices of preparedness as reflected in the operations of global and regional agencies, with a focus on the African continent, as well as in national institutions and selected rural villages in Sierra Leone and Uganda. Both countries are identified as “hotspots” and have relevant experience of Ebola that sharpened the interest of government and other agencies in preparedness. In particular, we have been concerned both with how the latest round of pandemic preparedness ideas are being mobilized in global and regional assemblages, and with (dis)connections to what we provisionally termed “preparedness from below” – the understandings and practices of people on the ground and how they prepare for and respond to exceptional or ongoing threats to health, life, and livelihood.

With the emergence of COVID-19, our project pivoted to the virus as a focus of fieldwork. This article shares findings related to the pandemic for the period up until the end of 2020, which provides a compelling case through which to ground and explore these concerns. Our team from August 2019 included research officers living in rural villages in Sierra Leone and Uganda, where they were able to continue their ethnographic fieldwork, albeit with consideration of COVID restrictions. Several team members have been participating in national, regional (in Dakar), and global COVID-19 committees and meetings in person and remotely. Drawing on fieldwork throughout 2020 (fieldnotes, informal discussions, observation, and interviews) and on available secondary literature, we begin by examining regional discourses and institutional responses across Africa. We then detail national government responses in Sierra Leone and Uganda, where in both contexts past experiences of outbreaks and international interventions as well as conflicts and political agendas have shaped planning and implementation of measures. Finally, we trace how directives were interpreted and experienced by rural villagers during 2020.

The picture is one of an uncertain and shifting situation during 2020. The image of a coherent global/regional preparedness assemblage (dis)articulating with national and local frameworks dissolves, and stages of preparedness and response emerge in practice as blurred. Amidst uncertainties about the epidemiological heterogeneity of the pandemic on the African continent, it is unsurprising that national messaging shifted and has been experienced as confusing and at times contradictory. It is evident that such uncertainties have influenced people’s decisions about whether and how to adhere to public health measures, and how they have reflected on and responded to these in diverse ways over time, in the light of personal experience, practical knowledge, competing health, livelihood and social priorities, and the ongoing precarities of rural life.

This leads us to expand the original concept of “preparedness from below” to encompass the “negotiation of intersecting precarities” in relation to epidemics, including those generated by insensitive public health responses. This dual conceptualization incorporates the concern in literature on precarity with states of insecurity and political exclusion as well as agency and contingent organization in response to such processes (Han 2018). It also enables a rethinking of epidemic preparedness and response as interconnected processes shaped by older and newly generated vulnerabilities and capabilities in navigating uncertainties, highlighting the critical value of ethnographic insights in understanding how these processes unfold in different settings.

## COVID-19 in Africa: Institutional framings and responses

In the wake of the West African Ebola experience and calls for greater investment, preparedness activities were initiated in several African countries in 2015–16, including efforts to expand JEEs across the continent and strengthen implementation of the International Health Regulations. By 2017, the Africa Centers for Disease Control and Prevention (ACDC) and its regional offices were inaugurated, with this African Union body leading plans for a laboratory network, to strengthen surveillance and early detection.

The emergence of COVID-19 led to inevitable anxiety regarding robustness of preparedness on the continent. In February 2020, an editorial by African infectious disease experts asked “is Africa prepared and equipped to deal with yet another outbreak of a highly infectious disease?,” whilst pointing out that 45% of countries had one epidemic annually (Kapata 2020: 233). As Egypt recorded the first case of the virus on the continent in mid-February, an analysis of which African countries were most vulnerable to a COVID-19 epidemic recorded worrying preparedness scores (Gilbert et al. 2020).

The WHO Africa office responded early and organized sub-regional meetings, gathering key stakeholders to define a roadmap for action, focused on better coordination and governance as identified following previous outbreaks. ACDC also played a key role in setting up joint operational platforms such as for continent-wide procurement mechanisms. ACDC convened an emergency meeting of health ministers on 22 February and an African Union Taskforce on Coronavirus began weekly meetings.^1^ ACDC worked to coordinate the expansion of scientific capacities such as testing (Maeda and Nkengasong 2021).

WHO Geneva made clear in a preparedness and response plan^2^ issued in early February that the novel coronavirus required extraordinary measures and prioritization, privileging in its guidance a response led by governments but emphasizing also the minimization of economic and social upheaval and concern for human rights.^3^ Having watched the Chinese state impose an unprecedented “lockdown,” European countries began instituting strict restrictions and COVID-19 was recognized as a pandemic in mid-March.

By the end of March, reported cases in Africa had risen to 3,700 and WHO advised a move from “readiness” to response, noting that previous epidemic experience would provide a basis for African action.^4^ Initial efforts focused on airport surveillance and quarantine of travelers. Several countries also initiated early and stringent “lockdown” measures with few detected cases, to buy time to prepare. At this time, there was wide concern that the virus would spread rapidly across the continent with devastating effect. Yet as 2020 progressed, the course of the epidemic on the continent confounded early models, and epidemiological curves based upon outbreaks elsewhere. It proved a source of significant debate, with heterogeneity across African country settings (Mbow et al. 2020). This unpredictable, diverse unfolding of the pandemic also began to challenge the notion of set phases of an epidemic in linear temporal progression, with acknowledgment that countries appeared to be in different phases.

From the outset there were challenges in interpreting the data regarding cases and deaths in Africa, given limitations in the scale of testing and death registration. However, even bearing this in mind, WHO accepted that the extent of mortality during 2020 had been lower than was expected, with 80% of cases estimated to have been asymptomatic.^5^ By the end of July 2020, WHO data indicated that the continent was edging to 1 million recorded cases and only 15,000 reported deaths, predominantly in four countries (South Africa, Egypt, Tunisia and Algeria), where first waves appeared to peak in July–August 2020. The low mortality overall in this first wave has been linked *inter alia* to demography, with younger and more dispersed populations, and possible innate population immunity from exposure to other viruses (eg. Nordling 2020). Debate about factors behind the “African paradox” has continued, with new inflections in 2021: detailed consideration is beyond the scope of this article. Of note, however, is that during the period of our focus in 2020, a narrative surfaced of African success in dealing with the pandemic, as well as ensuring a collaborative, continent-wide response effort (Maeda and Nkengasong 2021; Makoni 2020). One reading of the early measures in Africa is that the vertical, state-led model with lockdowns partly mirrored the responses adopted in Asia and Europe and advised by WHO, albeit implemented more swiftly. African scientists, ACDC and the WHO Africa office linked the early response to African experience^6^ in epidemics, with established measures such as tracing systems and border controls thought to have slowed the spread of the virus even if other measures were difficult to implement (Mbow et al. 2020). In early November, John Nkengasong, the director of ACDC, also praised on Twitter the strong leadership of the African Union in supporting responses of member states, criticizing early dire predictions in an interview.^7^ Some have argued that the lack of attention to the African success is yet another indication of the need to decolonize the gaze of global health (Büyüm et al. 2020).

Yet there are several caveats to a wholly positive account of the African experience during 2020. The first is that the effects of public health responses on economies, livelihoods, food security, and other health conditions have been devastating. The negative effects of “lockdown” restrictions manifested rapidly across the continent with economic impacts intensified by high reliance on informal livelihoods and markets for food (Barasa et al. 2020). The public health measures were instituted in the absence of formal social protection systems in most African countries. The extent of the precarity of livelihoods was simply not accounted for in preparedness plans. By May 2020, ACDC advised an easing of restrictions even while simultaneously acknowledging uncertainty as to whether the continent might yet become a frontier of the pandemic (Loembe et al. 2020). Politicization of the COVID-19 situation informs the second caveat for consideration of the African experience, although this phenomenon is hardly unique to Africa (Smith and Cheeseman 2020). There have been marked examples of human rights violations and militarization in some African settings (Levine and Manderson 2020; Parker et al. 2020) and indications that political distrust, fueled by corruption, aided spread of the virus (Ezeibe et al. 2020). A third caveat relates to the evident resource limitations. Observations in regional meetings in the early days of COVID-19 suggest that “anticipation” of the pandemic was not matched by attention to the lack of capacity for response, with a suggestion by one specialist that post-Ebola strengthening had encouraged a false sense of capacity. Major gaps emerged between policy guidelines and resource realities with respect to laboratory and clinical capacity, as well as standard preventive measures. Planning as if for Ebola is likely to have been a factor in the “exceptionalist” approach in the first period of COVID-19 preparedness, assuming a small number of cases treated by experts in specialist hospitals rather than a scenario of larger numbers requiring mobilization of primary services.

Notwithstanding these challenges, as 2020 drew to an end, the estimated cumulative mortality of 59,194^8^ across Africa remained low in the global tally, compared to the proportion of population represented by the continent. Yet uncertainty persisted regarding trajectories for the continent, as cases remained low in some countries whilst others saw a steady increase (The Lancet 2020). From late November there was intensifying concern about second waves and new variants in the most affected countries.^9^ Meanwhile, continental testing capacity remained low and seroprevalence studies suggested a far higher level of population exposure (Maeda and Nkengasong 2021). The new year brought new waves and a higher burden of mortality than in 2020.

Toward the end of 2020, the institutional focus of WHO and ACDC shifted discernibly toward “vaccine preparedness.” African experiences with COVID vaccines are beyond the scope and timescale of this article. Suffice to say that intensifying concerns about vaccine supply and access, as well as challenges to distribution, vaccine hesitancy, and vaccine evasion by viral variants, have all suggested that reliance on technologies alone might be premature for Africa (The Lancet 2020).

We now turn to our findings from Sierra Leone and Uganda to explore how preparedness and response plans played out in national contexts and in experiences of COVID-19 on the ground in two rural villages during 2020.

## Sierra Leone: responses and interpretations of COVID-19

The first known cases of COVID-19 in Sierra Leone arrived by air in late March 2020 but the country had been preparing for its arrival from late January. Measures were enacted by the relatively sophisticated epidemic preparedness and response governance structure which had been set up after the Ebola epidemic, receiving external investment. In January, the Emergency Operations Center (EOC), which became the National COVID-19 Emergency Response Center (NACOVERC) began to hold regular meetings to assess the risk and devise a response strategy, sending a delegation to assess the airport, instituting quarantines for arriving passengers and later halting flights. NACOVERC also started to prepare other “pillars” – case management, surveillance, risk communication – and to request financial assistance from development partners. An early speech by the President emphasized the dangers of COVID-19 and the importance of a national response. This made heavy reference to Ebola, with little acknowledgment of the differences between the diseases. There were regular press briefings by the President throughout subsequent months, and ongoing communication, mostly over television and radio. This extended to a song in Mende by singer Jay Shine. It presented COVID-19 as a severe disease from which everyone is at risk of death, without nuancing for COVID-specific vulnerabilities such as by age.

Control measures were imposed, although not as strictly as in some African countries. A three-day national lockdown started in the first week of April – a practice that had been used for Ebola, although then combined with house-to-house searches to identify sick people. This was eased but schools, places of worship, and some markets were closed, and travel restrictions and social distancing rules introduced. The capital Freetown saw the re-mobilization of measures familiar from Ebola, such as handwashing stations. The presence of the Chinese CDC, which invested significantly in Sierra Leone during and after Ebola, meant that some testing capacity was established early on, although focused in the capital. A Covid-19 treatment center was established at 34 Military Hospital, followed by Coronavirus Care Centers and Treatment Centers elsewhere in the district and later in other provincial headquarters. The first cases were in Freetown but as cases began to occur in other districts, they and their primary contacts – established through the listing system re-activated from the Ebola era – were also brought to the capital.

Our local fieldwork focused on the village of Tawoveihun^10^ (pseudonym), of Mende ethnicity, and in Moyamba District in the south, with interviews and observation also in neighboring markets and in the provincial headquarters of Bo. In terms of interpretations and understandings of COVID-19, a varied and shifting picture has emerged. Early in the outbreak, some people expressed fear and concern that this was a dangerous disease – perhaps even more frightening than Ebola because it was “in the air,” so everywhere. Some took national radio messages very seriously: as a woman in Bo said in April “this is a clear proof that Corona is a very dangerous disease, it doesn’t want to know your status, color, age or where you from. We must be careful of this virus.” Others, however, speculated that COVID-19 was a disease of Europe, the US and China, and not “real” for Sierra Leone. In Tawoveihun in April, for instance, a woman said: “I have no confidence over the existence of COVID-19. It is meant for the West. Ours was the Ebola.” Speculation circulated, as it had for Ebola, of diseases manufactured in the West, of sorcery and of disease spread through “witch planes.” Interpretations seemed less politicized than they had been for Ebola, which was sometimes cast as a conspiracy of the party then in power (the All People’s Congress, drawing its support from the north) toward the Southern and Eastern provinces. This may relate to the outcome of the latest elections which returned to power the Sierra Leone People’s Party, which has its electoral base in the south and east.

In April, a study indicated that a majority of villagers feared a disease with COVID-19-like characteristics more than one with Ebola-like features, with some explicit that this was because they knew how to protect themselves from Ebola (Kamara et al. 2020). However, fieldwork suggested changing perceptions of COVID-19 over time. As months went on, most people in Tawoveihun had not directly experienced the disease. In Bo, the prevalence of asymptomatic and mildly symptomatic cases seemed to be contributing to a gradual shift to a view that COVID-19 is “not serious” or “just a fresh cold” – if it existed at all.

By mid-August, the country tallied 1,940 confirmed cases and only 69 deaths.^11^ Levels of concern about COVID-19 seemed to be diminishing, and skepticism grew about what had initially been presented as an epidemic of a serious disease. A few sudden unexpected deaths, especially of older people, had occurred, but the absence of widespread COVID testing made confirmations slow and left causes ambiguous. In Tawoveihun some felt that the disease had not emerged; others that it would be around forever (like malaria) so they would live with it and continue their lives. People seemed unsure what to believe and turned most obviously to their direct experiences – which to date were mostly of a mild, or non-existent, disease. Nevertheless, others continued to look more widely and at broader implications, with anxiety. As one elder in Tawoveihun put it: “I’m worried about this illness, if it has brought everything to a halt in the world, how will Africans survive when they solely depend on the western world.”

In this context of uncertainty, confusion was inevitable. From April, villagers felt the reality of the COVID-19 “lockdown” as impacts on their lives and livelihoods, especially with respect to market closures and rising food prices. District lockdown produced many anomalies since people were cut off from major market centers according to the vagaries of colonial boundaries. Tawoveihun was cut off from Bo, its nearest market center, by military checkpoints. In the pre-harvest period, when interior villages like Tawoveihun are dependent on supplies of imported rice, this brought the prospect of hunger. Two women traders in Tawoveihun complained in April that “we are in a state of confusion about the little goods we have left on our [trading] tables – and how we can get to Bo for shopping when the roads are blocked.” These restrictions to wider trade were compounded by the closure in April of the local market at Mofombo. Some praised the government for acting so promptly and compared this to the delay of the previous government over Ebola (Richards et al. 2015). However, traders reported that the closing of the market had destroyed their livelihoods, so they had no choice but to resume trading. In June traders opened the market themselves “on their own authority,” in advance of the government easing restrictions.

The Paramount Chief instituted a by-law to prohibit intervillage movement which many declared problematic. Some felt that the Paramount Chief should have instituted stringent measures in villages bordering neighboring chiefdoms, but not everywhere. Despite the restrictions, villagers were able – and continued – to visit and work in their farms and gardens through the heavy periods of agricultural work – and growing hunger – to August. Similarly, while the government laid down rules about frequent handwashing and wearing of masks, these were largely ignored. Rules were not being enforced and there was no penalty. Such ignoring of COVID-19 control measures seemed, in large part, to reflect the fact that the disease ranked low, if at all, among people’s concerns at the height of the hunger season. Interviews about people’s main worries in August produced an array of pressing anxieties about food, health problems, children, and livelihood, but COVID-19 was never specifically mentioned. Confusion existed as to why the control measures for COVID-19 remained – albeit in a reduced form by August.

Villagers’ interpretations and practices may also reflect a legacy of distrust in local government, as well as in the health system. Despite post-Ebola strengthening efforts, deficiencies remain in health services and speak to a longer-term experience of unrealized promises. This limited the extent to which community health workers were able to deliver public health messages about COVID-19, let alone for these to be trusted. In rural villages, the system of chieftaincy is pervasive in all activities. Under Ebola diverse forms of public authority were a factor in determining whether or not response was effective. Tawoveihun is in a chiefdom in which local action, strongly led by the Paramount Chief, had ensured an absence of Ebola cases, even though surrounding chiefdoms had many. Fieldwork pre-COVID suggested that a crucial factor under Ebola was that everyone, including the chiefs, adhered to agreed rules. However, this is perceived to have subsequently slipped. Many villagers now offer “one law for you, another for me” as the reason for what they see as a lower post-Ebola trust. With COVID-19, ambiguities about the nature of the threat and laxness in the application of rules on markets and movement, seems to have undermined the discipline and trust that was evident during the Ebola response in this district.

In the last months of 2020, further variation emerged in how far rules were adhered to. As seen in practices and perspectives around wearing masks, ostensibly law in public spaces, this appeared to relate rather little to people’s concerns about a disease threat, and more to livelihood and authority concerns. Some villagers claimed to adhere, reiterating the message that masks protect from disease. Others admitted that they wore them only to avoid punishment; as a young man put it: “At night, all government NACOVERC staff go to nightclubs without masks. In the morning soldiers and police mount checkpoints flogging people to wear masks. I have to comply because they have power and if I don’t do it, I will be flogged.” While drivers of motorcycle taxis feared such check points especially, they also appreciated masks as shields against dust.

Official cases and deaths in Sierra Leone remained low in 2020 and into early 2021, although uncertainty remained. In December, an announcement^12^ was made from state house that curfews would be indefinitely suspended, a decision justified by the fact that the US CDC re-categorized the country as low risk. In early 2021, NACOVERC attention turned to the elite in Sierra Leone and the diaspora, targeting incoming air passengers. Thus, the national response circled back to focus on airport surveillance, while villagers continued to pursue their livelihoods amidst multiple uncertainties and precarities.

## National and local interpretations and responses: Uganda

In Uganda, the warnings about SARS-COV-2 arrived at a time of competing disease outbreaks across the country. Early meetings in the ministry of health revealed ongoing concern about yellow fever and typhoid, and also Ebola in Democratic Republic of Congo (DRC). A flurry of government meetings in late February indicated that COVID-19 was perceived as a genuine health threat for Uganda. The US CDC began assisting in response plans and provided two mobile diagnostic laboratories. Resource challenges were evident in testing capacity but also in a lack of masks nationally, with imposition of a strict control on existing supplies. The resource situation was complicated by the fact that the threat of a new epidemic coincided with a time when many donor-funded preparedness activities were ending. International NGOs working near the border with DRC were wrapping up projects in anticipation that the Ebola outbreak there would soon be under control.

Initially, efforts focused on surveillance at airports and borders. In March, broader containment measures were put in place with closure of schools and religious institutions, and restrictions on public transport and the sale of non-food items in markets. The government shifted COVID-19 planning to a dedicated national task force situated in the Presidency. What followed marked a decided militarization in the implementation of response, which gained Uganda international attention for the authoritarian nature of its “lockdown,” whilst at the same time the economic impacts on livelihoods became evident. To enforce measures, the government deployed the Local Defense Forces (LDUs) and the military. The LDUs are known for their brutality and indiscipline. The approach of implementing stringent presidential directives by forceful means was met with resistance in many parts of the country with people speculating that the state was using COVID-19 to reinforce its authority (Parker et al. 2020).

Early government messaging discussed COVID-19 as a virulent epidemic that had the potential to cause many deaths. However, by May no deaths had been reported and overall case numbers remained low. Trust in government initiatives, already low in Uganda, was further undermined. Public comment focused on the wastefulness of quarantining healthy people, especially as by June centers were struggling to process tests as laboratories ran out of reagents.

In our field village Buembe^13^ (pseudonym) in the district of Kasese on the border of DRC, Ebola has been a threat in recent years. Our research team noticed that health screening points for Ebola set up by a humanitarian organization were closed as the COVID-19 focus intensified in March, with diminishing attention to the possibility of Ebola resurfacing. Other infectious diseases were also on the priority list of district health authorities, such as a hepatitis B vaccination programme. As one villager commented: “We are overwhelmed with many diseases in this village. Before we finish this disease, another disease comes. We have not yet finished protecting ourselves from hepatitis B disease. I have heard that any time Corona will be coming in this village. This is becoming too much for us to understand.”

In Buembe, COVID-19 initially seemed very distant to many, spoken of as “a disease of the radio.” Names for COVID-19 in the early days in April and May included *obukoni obwasire* (“a disease that has come”) *obulwere obwabasungu* (“a disease that is associated with a white person”) and *obulwere* obwe China (“a disease from China”). However, some also articulated fear: one person indicated: “My fear of corona is very big. This radio tells me very many things. It tells me that very many people outside Uganda have died of corona. When I look at the number of people living in this village and compare them with the number of people outside Uganda who have died of corona, I just sit down, and more fear of the disease comes to my heart.” On the other hand, another reflected that they had not experienced COVID-19, saying: “The truth I can speak is that am not fearing corona very much because I do not know it. No one in this village knows it. I don’t see why the disease can be feared if it is not understood.” One person reflected on the origins of the disease as a man-made weapon in a distant geopolitical struggle, concluding, “ … the disease started to spread, and it may finally come into this village.” Comparing COVID-19 to Ebola, a man reflected that Ebola is “simple” and easy to deal with compared to the new disease which came with complicated instructions.

Observations from the villagers of politics at the national level began to draw comment and expressions of distrust in government claims and measures. When the “lockdown” measures were extended in May, one villager reflected that the president was looking to get money from foreign aid amidst corona threats, and to boost his political ambitions: “I have understood that the man of this country is bright. He is on the lookout of raising money for corona for himself and for his office in the name of fighting corona. He is pretending so that the coming elections become unclear to us.” The variation in opinion and uncertainty regarding how seriously to take the warnings was very evident as April moved into May. COVID-19 was not present as an experience of disease, but as a set of government restrictions enforced by a military presence. The public health messaging was received variably. Whilst handwashing and avoiding handshaking is familiar to people from Ebola-containment measures, villagers were unfamiliar with the idea of masks and unclear about the reasons for movement restrictions. The benefit of physical distancing was not immediately evident. In particular, people took issue with restrictions on funerals and the closure of churches, with some distressed that the government would close places of worship, a source of comfort. The messaging itself fueled theories about the disease, as people tried to interpret the reasoning behind measures. There was speculation in Buembe that if washing with a sanitizer made of alcohol is advocated, then drinking a locally brewed gin called *waragi* would be a cure. The imposition of a night curfew by government led to speculation that the disease spread nocturnally.

In the village, the authorities beat people in order to disperse them and prevent congregation in the market. People crossing the river border to work their fields in DRC were beaten and fined, fueling suspicions of corruption among officials, who were reported to demand payment to allow a bypassing of regulations. People also resorted to using informal paths to conduct trade in DRC, risking dangerous river crossings. Villagers experienced increasing livelihood difficulties because of restrictions in market trading and in accessing their fields and the river for water. As early as April, a woman trader described how “These days I am suffering because the source where I get money has been stopped. Even if I get anything to sell in the customs market, I cannot manage because the market is closed.” Rising food prices became a concern.

The measures enforced by the soldiers were viewed by villagers as excessive and out of touch with an understanding of people’s need to sustain their lives and livelihoods. Overall, the suffering from the restrictions exceeded any suffering from the disease, and this disparity became increasingly evident as the months passed but COVID-19 had not manifested in Buembe. In June, a villager expressed the prevalent view that the militarized implementation of the measures was not necessary: “Since I came on this earth, I have never heard that a disease can be watched using the gun. I don’t think a disease can be stopped by use of the gun. Corona is the example of the disease am speaking about.”

Local tensions with the military presence and distrust of state intentions extend back to a history of anti-government and anti-army feeling in this border district. People here look to alternative forms of public authority, including cross-border militias who shelter in Uganda. The perception of brutality manifested early on in local resistance to the military presence. Villagers invoked the spirit of the local river to reprimand the military commander who was seen to be over-extending his authority: “These soldiers think that this river valley is a simple place. They should not think that corona will make them manage this river valley … these soldiers reported the threat of the voice of the spirit to their commandant who also went there to prove. When night came, the spirit slapped the commandant. This is when he decided that his soldiers should watch the river valley up to a time that is not beyond 9 o’clock at night.”

With radio reports of the first deaths in Uganda in July, villagers expressed skepticism as to their truth as “corona” seemed to be more a political matter in the country. In July, the government stopped broadcasting primarily COVID-19 awareness messages, shifting to the indirect health impacts of the “lockdown” measures – urging ongoing malaria prevention and childhood vaccination. Yet COVID-19 measures such as a curfew remained in place. In August, other priorities arose in Buembe, such as severe floods that destroyed crops, hunger, and the resurfaced Ebola in DRC. People reported that they stopped listening to radio shows about COVID-19 disseminated by the District Task Force, tired of false alarms when villagers were dying of other diseases. Immediate tensions with the army eased, with some negotiating access to gardens.

In November reports circulated of cases in the district hospital; others claimed that they had not heard that people die of corona in their area. In December ACDC flagged Uganda as a country with a worrying uptick of cases. Official deaths remained low in early January 2021 at 301.^14^ As elections loomed in the new year, politicians were advised to conduct digital election campaigns. However, members of the ruling regime flouted COVID-19 measures whilst meetings organized by opposition groups were disrupted. Unease remained among scientists that a surge in cases might come, an eventuality that has come to pass in 2021 with the aid of further viral variants.

## Negotiating intersecting precarities

Amidst uncertainty about the epidemiological picture of COVID-19 in Africa in 2020 and the trajectory beyond, we have examined how preparedness and response, as interconnected processes, have been articulated and enacted in different settings through formal assemblages as well as informal mobilizations, including to mitigate the indirect effects of public health measures.

Consideration of the diverse unfolding of COVID-19 in Africa over 2020 challenges notions of a standardized response to a pandemic across the continent, with set progression through stages of emergency in a linear temporal frame. Government responses in African settings in 2020 were instituted early, often prior to significant viral spread, such that people were interpreting public health messages and navigating restrictions in the absence of experience of the actual disease. COVID-19 itself has emerged as a longer-term issue, with recurring waves over the months but growing consensus that it is unlikely to disappear. A clear temporal distinction in the emergency cycle between the stages of preparedness and response is called into question by the experience of preemptive responses, unpredictable waves of infection and warnings of endemicity.

The heterogeneity of the epidemic, and the limitations in resources and capacities, bring further uncertainty in “the science,” as insufficient testing capacity continues to erode definitive knowing. Furthermore, the stringent measures which are credited with flattening epidemiological curves in the earlier part of 2020 have been ruinous economically and caused considerable suffering for people living in circumstances of precarity, a challenge which has recurred as further restrictions have attempted to control subsequent waves into 2021. A vertical “emergency” framing of health crisis and the resultant disease-focused health security response has also affected healthcare for other prevalent conditions. Unsurprisingly, greater adaptation of standardized approaches and more contextually sensitive public health and mitigation measures would have been preferable. National responses have not just followed the epidemiology and the (uncertain) science and unexpected temporalities but are also being shaped by political history and experiences of previous outbreaks. Arguably, scientific uncertainty makes space for more political influence, not less.

Uncertainty plays out in the confusion on the ground in terms of how people try to make sense of the new disease over time. As government messages shifted or seemed at odds with people’s own observations, skepticism grew in some quarters. People’s reflections in both contexts during 2020 point to the relevance of direct experience of disease, of “seeing” it for oneself or knowing someone who has had it. The idea of COVID-19 as a foreign disease in the early stages of COVID-19 has been reported also from other African settings (Geissler and Prince 2020a) and in our fieldsites was strengthened in the absence of such direct knowing or seeing in the villages. Such experiential knowledge seems more significant to people’s decisions than the official expertise of state agencies, or health workers. Radio has been important in both cases as a source of information, but its messages act more to distance and displace since they do not reflect everyday experience or priorities; a disease becomes of the radio, or of foreign places – not one to be negotiated in one’s everyday life.

Political dynamics are evident in both countries, as well as the role of other forms of public authority in villages. We have seen local politics unfolding in and through the response, whether in the contestation between diverse forms of public authority in Uganda (militias, the LDUs), or in the interplay between chiefly authority and other forms of agency and allegiance, such as amongst market traders, in rural Sierra Leone.

In both countries the importance of past experience of Ebola is evident; people’s understandings of COVID-19 are to some extent comparative. The shaping of responses to COVID-19 by past and ongoing experiences of epidemics has been noted elsewhere on the continent (Geissler and Prince 2020b). With time, comments emerged about other diseases as seemingly more important. Other competing priorities have also been very present, from floods to hunger and concerns with livelihoods, with some of these pressing issues worsened by seemingly purposeless restrictions related to COVID-19. People in both villages are navigating the potential threat of COVID-19 amidst a host of everyday precarities and often finding that the pandemic itself is not an immediate priority, although the restrictions have exacerbated their problems. In these circumstances, there was little evidence during 2020 of village-level responses to protect people from COVID-19 infection, or “preparedness/response from below” directed to the disease itself, as was observed in villages in Sierra Leone with Ebola. Rather, people have been trying to navigate the restrictions imposed to prevent COVID-19 transmission. In both villages we see people finding means to bypass rules – whether through bribes or a re-opening of markets despite official prohibitions. The negative effects of the “lockdowns” imposed over many months on their lives and livelihoods, in the absence of experience of the actual disease, has been perceived in both contexts as the more immediate threat requiring local action, negotiation, and sometimes resistance, whether organized (eg. in militias or trader groups) or more ad hoc and contingent. Insensitive public health and epidemic control efforts have in both settings intersected with, and sometimes intensified, existing precarities related to hunger, poverty, and social and political vulnerability. These are part of the ongoing substrate of the “slow emergencies” (Anderson et al 2019) that people are familiar with and which compete with a pandemic temporality that prioritizes one anticipated crisis above all others.

It has been argued that the effects of COVID-19 in many parts of the globe are intensifying inequalities and surfacing the intersecting nature of crises (Dowler 2020). Drawing on this idea, our findings advance and illustrate the concept of “intersecting precarities” which includes the effects of epidemic control measures themselves. We contend that people do not just accept but actively negotiate these intersections as they seek to sustain their lives and livelihoods, including through mutuality and collective action. Having the preexisting capabilities, forms of collective organization, and experiences in dealing with everyday uncertainties to do so, is key to “preparedness from below.” The concept of precarity is salient here as it incorporates consideration of ontological states of insecurity as well as the ways in which political-economic processes engender states of exclusion as they play out in particular localities (Han 2018), providing an articulation of how forms of vulnerability have been revealed and have coalesced as a consequence of the pandemic. A concern with the pandemic’s intensification of preexisting conditions of life also moves us beyond the image of a discrete event and temporality such as an outbreak to address the unfolding of precarities – acute crises compound chronic uncertainties that might be of greater local priority, and a pandemic threatens to introduce yet another endemic challenge that must be lived with and navigated in the present. A foregrounding of intersecting precarities also underscores the importance of expanding formal understandings of preparedness to include attention to systemic conditions that influence the effectiveness of response – the “staff, stuff, space and systems” that Farmer (2014) so pertinently highlighted as the necessary substrate for effective epidemic control.

## Conclusion

ACDC and WHO swiveled in late 2020 to vaccines as the continent faced the uncertainty of a second wave of COVID-19. However, the ongoing structural inequalities have persisted, as have the limitations in resources to respond to an outbreak or to marshal the social and material infrastructures required to deliver technological solutions in countries like Sierra Leone (Lee et al. 2020). The experience of COVID-19 might concretize an authoritarian and top-down vision of preparedness and response (Caduff 2020) that limits examination of conditions that drive disease emergence, and structures of power that undermine preparedness and profit from the inequalities that epidemics exacerbate (Erikson 2020). Yet there is also an opportunity to foreground the actions of ordinary people, and to appreciate that epidemiological constructs of disease origin and spread can be at odds with ontological positions that privilege explanations oriented to why misfortune has manifested at a given moment and how to address present circumstances (Lynteris 2020). The embodied uncertainties navigated in the present can be more salient than preparing for an unknown future event predicted by an authority that lacks trust or appears remote and unconcerned about everyday realities.

Whilst we have explored these issues in two African countries with relatively low numbers of COVID-19 cases and deaths, at least during 2020, our findings and analysis have broader significance including to settings with very different disease experiences. A consideration of intersecting precarities and preparedness from below together, in a dual conceptualization, helps to capture the dynamics and timeframes at stake in epidemics more effectively than do linear separations of “preparedness” and “response.” Attending to “the below” foregrounds the significance of local forms of knowledge, experience, and agency in navigating uncertainties. These dimensions are important whatever the epidemiological picture. In this light, epidemic preparedness and response cannot simply involve attempts to foresee and control risk of disease, through standardized, largely technical processes. Rather, it requires appreciation of preparedness and response as interconnected processes, and of multiple uncertainties and complex effects and how people interpret and respond to them, amidst everyday precarities. As the multiple experiences of COVID-19 continue to catalyze global debates, we can hope that they incorporate greater appreciation of these dynamics, and thus more sensitive approaches attentive to unfolding local experiences and priorities.

## Notes

1. HM and ML led on conceptualization and writing. The remaining coauthors are from the Pandemic Preparedness project and listed alphabetically. They contributed text to specific sections, supervised fieldwork or conducted data collection.

2. WHO, 2020, Strategic plan. https://www.who.int/publications/i/item/strategic-preparedness-and-response-plan-for-the-new-coronavirus.

3. WHO, 2020, Director-General’s remarks 11 March 2020. https://www.who.int/dg/speeches/detail/who-director-general-s-opening-remarks-at-the-media-briefing-on-covid-19—11-march-2020.

4. WHO, 2020, African Region numbers. https://www.afro.who.int/health-topics/coronavirus-covid-19.

5. WHO, 2020, Africa’s low COVID-19 cases. https://www.afro.who.int/news/social-environmental-factors-seen-behind-africas-low-covid-19-cases.

6. Bearak, Max and Paquette, Danielle, 2020, The coronavirus is ravaging the world. Washington Post, December, 11. https://www.washingtonpost.com/world/africa/africa-coronavirus-low-cases-deaths/2020/12/10/e907a1c2-3899-11eb-aad9-8959227280c4_story.html.

7. Brown, Will, 2020, “A shallow understanding of the continent”. The Telegraph, October, 9. https://www.telegraph.co.uk/global-health/science-and-disease/shallow-understanding-continent-africa-has-defied-covid-19-expectations.

8. Johns Hopkins Coronavirus Resource Center. https://coronavirus.jhu.edu/.

9. WHO Africa, 2020, African countries see steady COVID-19 rise. https://www.afro.who.int/news/stronger-action-needed-african-countries-see-steady-covid-19-rise.

10. The main livelihood activities are farming, fishing, charcoal burning, trading, and *okada* (bike) taxi riding. The farming is small-scale subsistence; two or three months after harvest farmers will need to buy rice again. A few people have cash crop plantations such as coffee, cocoa, kola nut, and palm oil. There is also cattle-rearing.

11. Johns Hopkins Coronavirus Resource Center. https://coronavirus.jhu.edu/.

12. Sierra Leone Government, 2020, Public Notice, November 27. http://dhse.gov.sl/wp-content/uploads/2020/11/PRESS-RELEASE-3.pdf.

13. The main livelihood activities include domestic livestock and crops such as maize, sweet-potato and cassava. Surplus is sold in markets to cover school fees, clothing and medicine. Some people have cash crops including coffee and cocoa. Construction work, motorcycle transport, sand mining in the river, charcoal and brickmaking are other sources of income.

14. Johns Hopkins Coronavirus Resource Center. https://coronavirus.jhu.edu/region/Uganda.
